# Supplementary material for: DMRforPairs: identifying Differentially Methylated Regions between unique samples using array based methylation profiles
Source: BMC Bioinformatics. 2014 May 15;15:141. doi: 10.1186/1471-2105-15-141 (PMC4046028; doi:10.1186/1471-2105-15-141)
Supplement: Additional file 2 — DMRforPairs output for the comparison of A431-MCF7 and NA17018-NA17105. Please start from the HTML files in each folder. Available via the BMC Bioinformatics website. [file 1471-2105-15-141-S2.zip › 1394847754114233_MOESM2_ESM/A431_MCF7/figures/34434.pdf]

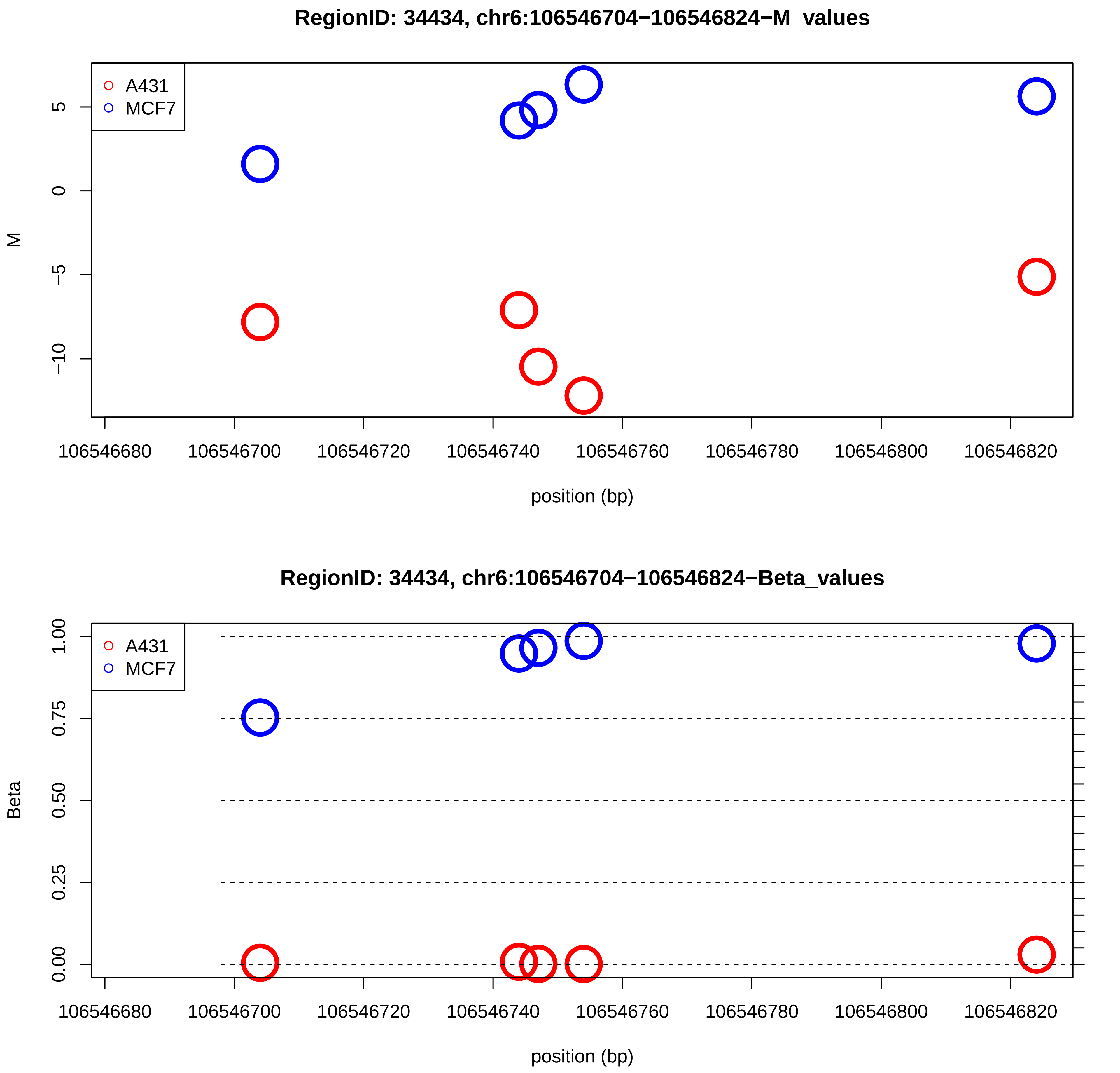
RegionID: 34434, chr6:106546704−106546824−M_values
A431
MCF7
M
5
0
−5
−10
106546680
106546700
106546720
106546740
106546760
106546780
106546800
106546820
position (bp)
RegionID: 34434, chr6:106546704−106546824−Beta_values
A431
MCF7
Beta
1.00
0.75
0.50
0.25
0.00
106546680
106546700
106546720
106546740
106546760
106546780
106546800
106546820
position (bp)

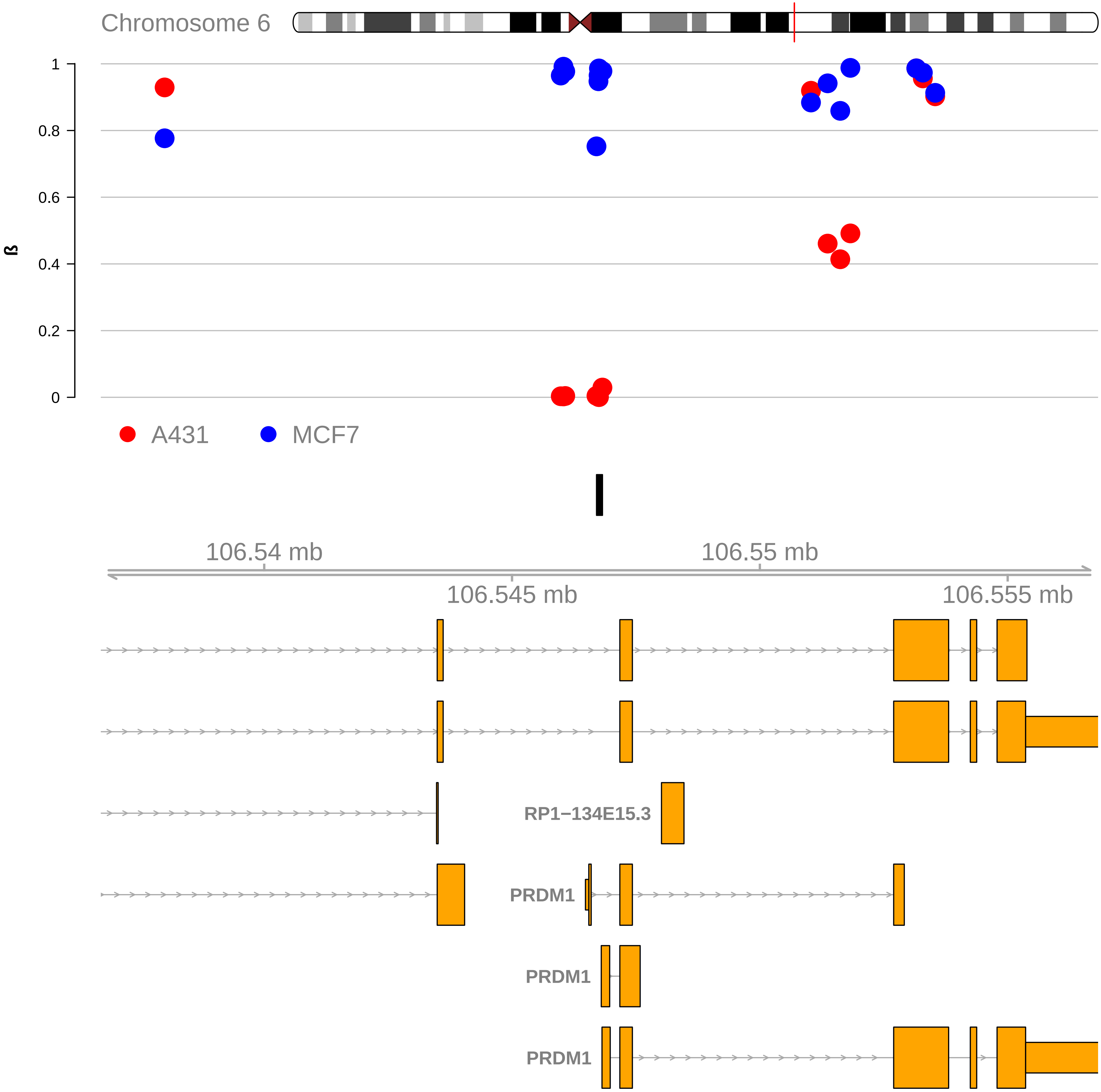

Chromosome 6
1
0.8
0.6
0.4
0.2
0
ß
A431
MCF7
106.54 mb
106.55 mb
106.545 mb
106.555 mb
RP1−134E15.3
PRDM1
PRDM1
PRDM1
